# Enhancing the texture and nutritional value of pumpkin dessert/jam through vacuum impregnation pre‐treatment with calcium and vitamin D3

**DOI:** 10.1002/fsn3.4122

**Published:** 2024-04-02

**Authors:** Elif Buse Taş, Oguz Gursoy, Yusuf Yilmaz

**Affiliations:** ^1^ Graduate School of Natural and Applied Sciences, Division of Food Engineering Burdur Mehmet Akif Ersoy University Burdur Turkey; ^2^ Department of Food Engineering, Faculty of Engineering and Architecture Burdur Mehmet Akif Ersoy University Burdur Turkey

**Keywords:** calcium oxide, impregnation, pumpkin, vacuum impregnation, vitamin D3

## Abstract

This study involved fortifying pumpkin slices with calcium and vitamin D3 using vacuum impregnation (VI) pre‐treatment and assessing the quality characteristics of the resulting desserts/jams. Slices were subjected to immersion or VI pre‐treatments for 30, 60, and 90 min in a solution containing calcium oxide and vitamin D3. Calcium ions contributed to the hardness of desserts, with VI reducing processing time. The highest impregnated calcium (58.17 mg/100 g fw) and vitamin D3 contents (6.02 mg/100 g dm) were determined in slices pre‐treated by VI for 90 min. VI was more effective than immersion in terms of calcium and vitamin D3 transition into pumpkin tissues. Scanning electron microscope (SEM) images indicated that calcium oxide particles were noticeable in slices pre‐treated by VI. Immersing fruit slices for 90 min produced desserts with a textural hardness of 11.04 N, while VI pre‐treatment for the same duration increased their hardness value to 18.92 N. Desserts produced with VI‐pre‐treated slices exhibited superior texture and sensory attributes, with no adverse taste resulting from calcium oxide. In conclusion, VI pre‐treatment shows significant potential for the industrial production of desserts/jams with enhanced structural integrity for fruits.

## Graphical abstract

The infusion of calcium and vitamin D3 into pumpkin slices was more efficient and faster with the use of vacuum impregnation pre‐treatment compared to immersion. The incorporation of calcium and vitamin D3 into fruit slices enhanced the texture and nutritional value of pumpkin dessert, while the use of calcium oxide did not introduce any unpleasant taste. This suggests that this pre‐treatment holds significant promise for industrial applications in the production of traditional pumpkin desserts with enhanced structural integrity.

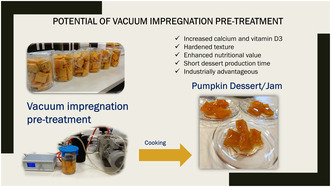

## 1 INTRODUCTION

Cucurbits are part of the Cucurbitaceae family and include 118 genera with 825 species in their taxonomic classification (Jeffrey, 1990), and *Cucurbita maxima* Duchesne, commonly known as the pumpkin, belongs to this family. According to FAO (2018), global pumpkin production exceeded 30 million tons in 2022, while almost half of this was produced by China and India, which were followed by Russia, Ukraine, the USA, and Mexico. Pumpkin fruits are important for human nutrition because fresh pumpkin fruits with a dry matter content between 7% and 10% are highly rich in fiber, carotenoids (especially β‐carotene), and minerals like calcium and potassium but very low in sodium (Guiné et al., 2012). Pumpkin fruits can be consumed through various preparation methods and culinary processes, and can be used especially for making desserts, jams, and soups. The skin of its fruits is hard, usually gray‐orange, and the interior is soft and yellow‐orange compared to the peel. Pumpkin fruits contain lipophilic carotenoids, which are responsible for the orange color of fruits, and carotenoids may increase the absorption of vitamin A, which is essential for the development of vision, embryo development, and growth (Seoa et al., 2005).

Osmotic dehydration serves as a pre‐treatment for fruits and vegetables before undergoing preservation methods like freeze‐drying, vacuum‐drying, microwave‐drying, air‐drying, and freezing (Fito, 1994; Silva et al., 2014). The purpose of osmotic dehydration as a pre‐treatment is to increase the nutritional value of foods, minimize any loss in nutritional value and aroma of foods during processing, improve the functional properties of foods, increase the shelf life of foods, and save energy. While osmotic dehydration is generally applied under atmospheric pressure, vacuum impregnation (VI) involves applying a reduced pressure (vacuum) to accelerate the rate of osmotic dehydration (Silva et al., 2014). The VI method aims to modify the chemical composition of food products by partial dehydration and/or impregnation by transferring soluble substances without affecting the structural integrity of the products (Fito, 1994). The factors influencing VI are typically categorized under internal and external factors, and the former includes the type, texture, shape, and size of fruits and vegetables, while the latter includes the type, concentration, composition, and temperature of the impregnation solution, the type and molecular weight of the solvent used as impregnation solution, vacuum pressure and duration, atmospheric pressure restoration if used, the mixing process, and finally the ratio of the solution to the sample (Yilmaz & Ersus Bilek, 2017).

Softening in fruit tissues may alter the cell wall structure, cause the loss of cell wall integrity, and breakdown cell wall components, ultimately leading to cell separation. Calcium ions play diverse roles in plant physiology, especially in maintaining the stability of the cell wall and membrane by binding to the pectin polymer in the middle layer of the cell wall (Hocking et al., 2016). Treating fruits with calcium has been shown to delay ripening by preserving membrane integrity and enhancing cell wall firmness (Zhi et al., 2017). Numerous studies have demonstrated that calcium treatment can suppress the expression and activity of cell wall‐degrading enzymes, decelerate the depolymerization of pectin and hemicellulose polysaccharides, thereby preserving textural quality and extending the shelf life of fruits and vegetables (Ayón‐Reyna et al., 2017; Liu et al., 2022; Lu et al., 2018; Muengkaew et al., 2018). Calcium oxide (CaO) and calcium chloride (CaCl_2_) are two primary sources of calcium ions utilized widely to improve the textural quality of fruits. CaO is obtained by heating calcium carbonate at around 900–1000°C. At room temperature, it exists as white, solid alkaline crystals, which are odorless. Upon reacting with water, it transforms into calcium hydroxide (Ca(OH)_2_). CaO (E529) is a food additive permitted for use under good manufacturing practices. It serves as an acidity regulator or a flour processing agent in a variety of food products.

Certain fruit jams or desserts are traditionally made from fruits that are vulnerable to the high temperatures involved in the cooking process of production. When subjected to elevated temperatures, the cell integrity of these fruits is compromised, resulting in a non‐particulated product that is undesirable for consumers. To prepare the traditional jams of green tomatoes, pumpkin, eggplants, peels of watermelon, and fig, 200–500 g of quicklime, primarily composed of calcium oxide (CaO), is initially dissolved in water for about 24 hours. Subsequently, the powdery component is allowed to settle down at the bottom. The clarified top part is used as an immersion solution for fruit slices. In the case of traditional pumpkin dessert/jam, sliced pumpkin fruits are kept in this solution for about 12–18 h. Then, syrup containing sucrose and water (1:1, w:w) is usually added to pumpkin slices. Finally, this mixture is cooked, preferably on a stove with low heat, for about 2–3 h.

Calcium and vitamin D are two complimentary nutrients for maintaining human health, and each has a very diverse function in the body. Calcium is the most prevalent mineral in the human body. Maintaining a proper calcium balance is crucial in the human body, dependent on the interplay between calcium intake and absorption on one side and urine excretion on the other. Over the long term, insufficient calcium intake has been observed to lead to various disorders such as osteoporosis, osteomalacia, and bone fractures, particularly in elderly individuals. In the US food supply, 73% of calcium intake comes from milk products, 9% from fruits and vegetables, 5% from grain products, and the remaining 12% from other sources (CNPP, 1996). Vitamin D2 (ergocalciferol, ergosterol) and D3 (cholecalciferol) are the most important vitamins among compounds exhibiting vitamin D activity. Ergocalciferol is derived from ergosterol and is found in plants; however, it is a less commonly utilized form for fortification purposes. Ultraviolet B (UVB) rays are essential for the synthesis of vitamin D in individuals, and cholecalciferol can be both synthesized within the human body and obtained through dietary sources. In the liver, cholecalciferol and ergocalciferol are converted to calcifediol (25‐hydroxycholecalciferol) and 25‐hydroxyergocalciferol, respectively. Calcifediol is further hydroxylated by the kidneys and some of the immune system cells to form calcitriol (also known as 1,25‐dihydroxycholecalciferol), the biologically active form of vitamin D (Holick, 2007). Excessive exposure to sunlight does not lead to vitamin D intoxication, as the excess vitamin D synthesized in the skin is inactivated by sunlight and converted into inactive products (FAO, 2002). The majority of vitamin D (80%–100%) is stored in the fat tissues located in the lower layer of the skin in the human body, and factors such as skin color, age, exposure time, and the angle of sunlight are influential in the synthesis of vitamin D in the lower layers of the epidermis (FAO, 2002). Vitamin D stored in fat tissues is released into the circulation when needed, and in the case of a vitamin D‐deficient diet, only 10%–15% of calcium and 60% of phosphorus can be absorbed, while this ratio increases to 30%–40% for calcium and 80% for phosphorus in the presence of vitamin D in a diet (Holick, 2007). Simultaneously, vitamin D serves various functions in the body, including reducing calcium excretion from the kidneys, decreasing parathormone synthesis and release, promoting bone resorption, enhancing insulin production, increasing myocardial contractility, and decreasing renin synthesis (FAO, 2002; Holick, 2007). Moreover, Shah et al. (2021) reported that the dietary intake of vitamin D at 5.25 μg/day significantly decreased the risk of type 2 diabetes mellitus by 21% in men, and a significant association between dietary calcium intake (812 mg/day) and this type of diabetes was observed in women.

In this study, two different pre‐treatments, immersion under atmospheric pressure and VI under 100 mbar, were employed to fortify pumpkin slices with calcium and vitamin D3. The application of vacuum facilitated the accelerated infusion of calcium into the slices during the production of traditional pumpkin desserts/jams with hardened layers. CaO (0.15%, w/v) and vitamin D3 (0.10%, w/v) were incorporated into the impregnation solution to enhance the nutritional value of pumpkin desserts/jams. It is noteworthy that, to the best of our knowledge, VI pre‐treatment has not been previously applied for the impregnation of calcium and vitamin D3 into pumpkin slices. This innovative approach could be industrially used in the production of pumpkin desserts or jams featuring a hardened outer layer.

## 2 MATERIALS AND METHODS

### 2.1 Materials

Pumpkins from the same field were obtained from a local producer in the city of Burdur (Turkey). Pumpkins, known as winter fruits, were purchased in December 2019 and January 2020 and stored at 4 ± 1°C before processing. The CaO and vitamin D3 chemicals were obtained from Tekkim Chemical Industry Trade Ltd. Co. (Bursa, Turkey) and CCPA Turkey's Animal Feeding Products Inc. (İzmir, Turkey), respectively. Trolox®, sodium carbonate, NaOH, AlCl_3_·6H_2_O, and chromatographic grade methanol were purchased from Sigma‐Aldrich (St. Louis, MO, USA), while Folin–Ciocalteu reagent and chromatographic grade ethanol were obtained from Merck (Darmstadt, Germany). 1,1–2,2′‐Azino‐bis (3‐ethylbenzothiazoline‐6‐sulfonic acid) (ABTS) was obtained from Fluka (Kenilworth, NJ, USA).

### 2.2 Preparation of pumpkin slices

Fresh pumpkin fruits were carefully peeled and sliced using a sharp knife, resulting in slices with approximate dimensions of 40 × 40 × 10 mm. The slices of pumpkin fruit were randomly divided into six groups, each weighing 500 g (one replication). All experiments were carried out in triplicate.

### 2.3 Immersion and vacuum impregnation pre‐treatments

The fruit‐to‐solution ratio for pumpkin fruit slices to impregnation solution was set at 1:2 (w/v). CaO (1.5 g, solubility 0.16%) and vitamin D3 (1.0 g) were combined with distilled water (1 L). To enhance the solubility of CaO, a mechanical homogenizer (WiseTis® HG‐15D, Daihan Scientific Co. Ltd., South Korea) was used at 12,000 rpm for 3 min. Subsequently, pumpkin slices (500 g) were added to this solution and subjected to various pre‐treatments.

**FIGURE 1:**
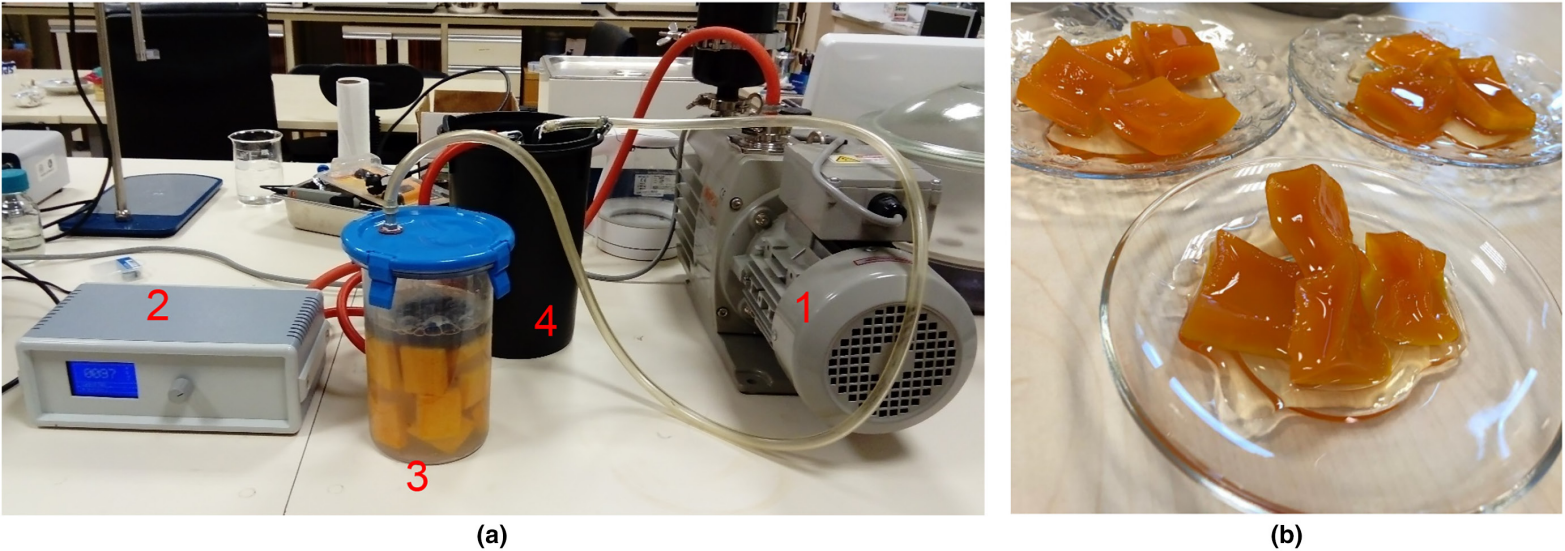
Assembly of vacuum impregnation unit (1: vacuum pump, 2: vacuum controller, 3: vacuum container and 4: liquid trap) (a) and an image of pumpkin desserts produced with pre‐treated pumpkin slices (b).

Pumpkin fruit slices underwent immersion pre‐treatment at room temperature under atmospheric pressure for durations of 30, 60, and 90 min. To ensure the slices remained submerged throughout the process, a galvanized wire and a weight ring were used. Following the pre‐treatment, the pumpkin slices were carefully removed from the solution and placed in distilled water for 10 min to eliminate any residual solution traces. VI pre‐treatment was carried out at room temperature under vacuum pressure (100 mbar). The process involved a vacuum pump (MVP 24, Woosung Vacuum Co. Ltd, Jeju, South Korea), a vacuum pressure controller unit, and a vacuum container (RV‐261, Re‐Va, Izmir, Turkey). This procedure took place in the Research Laboratory of the Department of Food Engineering at Burdur Mehmet Akif Ersoy University in Turkey. Vacuum impregnated pumpkin slices were kept in distilled water for 10 min to remove any traces of impregnation solution. The VI unit is shown in Figure 1a.

Following the pre‐treatments, the pumpkin slices were stored in a refrigerator at 4 ± 1°C. Similarly, dessert samples were refrigerated after being cooled to room temperature.

### 2.4 Dessert‐/jam‐making process

After the pre‐treatments, a portion of the pumpkin slices was set aside for chemical and physico‐chemical analyses, while the remainder was utilized in the production of desserts. The proportions of fruit, sucrose, and distilled water were maintained at 4.0:2.0:2.5 (w/w/w). Consistently, the same stainless‐steel pot (with a diameter of 16 cm and a volume of 1.5 L) (Sofram, Istanbul, Turkey) and an LPG‐fueled burner (with a diameter of 51.3 mm) (Çetintaş 303, Istanbul, Turkey) were used in all experiments. During the cooking process, sucrose was initially dissolved in distilled water for 15 min, then mixed with pumpkin slices, and all were cooked on the lowest setting of the burner for 85 min. The visual representation of pumpkin desserts/jams is shown in Figure 1b.

### 2.5 Dry matter, soluble solids, and ash contents

Pumpkin samples were initially grated and subsequently homogenized using a mortar and pestle. Approximately 20 grams of samples were then dried in an oven at 75°C (EN500, Nüve, Ankara, Turkey) until a constant weight was reached. The dry matter contents of the samples were determined gravimetrically according to the procedure of the AOAC (1990).

To determine their soluble solid contents (°Brix), grated and crushed samples (5 g) were mixed with distilled water (50 mL) and homogenized by a homogenizer (WiseTis® HG‐15D from Daihan Scientific Co. Ltd., South Korea). Then, a portion of this mixture was centrifuged in Eppendorf® tubes by a microcentrifuge at 12,225g for 15 min. The °Brix values of the clear supernatants were finally determined by a refractometer (PAL‐3 model, Atago, Tokyo, Japan).

For the determination of ash contents, dried samples (about 3–5 g) were weighed into crucibles and incinerated at 550°C for 18–24 h until their color turned gray‐white in a furnace (Protherm Furnaces PLF 110/6, Ankara, Turkey). The ash content of samples was determined according to the procedure of the AOAC (1990).

### 2.6 Determination of water activity and pH

The water activity of grated and crushed samples was determined at room temperature directly by a water activity‐meter (Testo 645, Testo SE & Co. KGaA, Lenzkirch, Germany) using the equilibrium relative humidity (ERH%). To determine the pH of samples, grated and crushed samples (5 g) were first mixed with 50 mL of distilled water and then homogenized by a homogenizer (WiseTis® HG‐15D, Daihan Scientific Co. Ltd., South Korea). The pH of these mixtures was determined by an immersion‐type pH‐meter (Testo 205, Testo SE & Co. KGaA, Lenzkirch, Germany) that had been calibrated with buffer solutions at pH 4.0 and 7.0.

### 2.7 Mineral content

The mineral contents of pumpkin samples were determined by an inductively coupled plasma/optical emission spectroscopy (ICP/OES) device (Optima 800, Perkin Elmer Inc., Waltham, MA, USA) according to the procedure of Altundag and Tuzen (2011).

### 2.8 Vitamin D3 content

The vitamin D3 contents of pumpkin samples were determined using an HPLC unit (Shimadzu Prominence, Shimadzu Corp., Kyoto, Japan) equipped with an SPD‐M20A detector, a C18 column (250 × 4.6 mm, 5 micron), a CTO‐10ASVp column oven, an LC20AT pump, a SIL20ACHT autosampler, and LC Solution software. The mobile phase consisted of acetonitrile:methanol:water (60:25:15 by volume). Prior to analysis, the pumpkin samples were freeze‐dried. Dried samples (1 g) were extracted with the mobile phase following the method described by Karppi et al. (2008). The vitamin D3 contents were determined at a wavelength of 200 nm.

### 2.9 Extraction of bioactive constituents

Extracts were prepared for both untreated and pre‐treated pumpkin samples, as well as pumpkin desserts/jams. The pumpkin samples were initially grated and crushed with a mortar and pestle. Portions of the homogenized pumpkin samples (5 g) were mixed with 50 mL of aqueous ethanol (70%, v/v) and homogenized by a homogenizer (WiseTis® HG‐15D, Daihan Scientific Co. Ltd., South Korea) at 1 for 2 min. This mixture was ultrasonicated for 10 min in an ultrasonic water bath (Wise Clean Wisd WUC‐D06H, Daihan Scientific Co. Ltd., South Korea). Subsequently, the mixture was transferred into an Eppendorf® tube and centrifuged at 13,500 rpm for 15 min. The clear supernatants obtained from this process were stored at 4 ± 1°C in a refrigerator.

### 2.10 Total phenolic content

The ‘micro‐adapted Folin–Ciocalteu’ (FC) method was used to determine the total phenolic contents (TPCs) of samples (Vernon et al., 1999) by using a UV–Vis spectrophotometer with a rotary type 8 position multi‐cell holder (Optizen Pop, Mecasys Co., Ltd., Daejeon, South Korea). The TPCs of the samples were expressed in gallic acid equivalent (GAE)/100 g dm.

### 2.11 Total flavonoid content

Total flavonoid content (TFC) analysis was performed according to the method of Zhishen et al. (1999). Catechin (20–100 mg/L) was used to obtain a calibration curve, and results were expressed as catechin equivalent (CE)/100 g dm.

### 2.12 Antioxidant capacity by the ABTS method

ABTS radicals (ABTS•+) were generated by dissolving ABTS in distilled water and combining it with 2.45 mM potassium persulfate at a 1:1 (v/v) ratio. This mixture was allowed to stand at room temperature for 12–16 h, and then diluted with chromatographic grade methanol to achieve a final absorbance of 1.20 ± 0.02. The ABTS assay, as described by Thaipong et al. (2006), was used to determine the antioxidant activity (AA) of samples. The AA values of the samples were expressed in μmol Trolox® equivalent (TE)/g dm.

### 2.13 Color measurements

Color measurements were conducted by a colorimeter (CR400, Konica‐Minolta, Osaka, Japan). Grated pumpkin samples were finely crushed using a mortar and pestle, and homogeneous samples were transferred into an optical glass cell (34 mm diameter) provided by the manufacturer of the colorimeter. The color values of samples were expressed on the scale of CIELAB (Commission International de L'Eclairage), where L* represents lightness (0 = black and 100 = white), a* indicates redness‐greenness and b* indicates yellowness‐blueness. Color measurements were taken using specular reflection included with the D65 illuminator, a 10° observer angle, and an 8‐mm aperture. Prior to color measurements, the colorimeter was calibrated using a white standard plate, and total color difference (Δ*E*), total chroma difference (Δ*C*), color index (CI), chroma (*C*), and hue angle values were determined (Karacaoglu et al., 2016) by using the Equations ((1), (2), (3), (4), (5)):

(1) $$∆E=\sqrt{{\left(∆L^{*}\right)}^{2}+{\left(∆a^{*}\right)}^{2}+{\left(∆b^{*}\right)}^{2}}$$

(2) $$∆C=\sqrt{{\left(∆a^{*}\right)}^{2}+{\left(∆b^{*}\right)}^{2}}$$

(3) $$C=\sqrt{{a^{*}}^{2}+{b^{*}}^{2}}$$

(4) $$\mathrm{Hue}\;\text{Angle}=\tan^{-1}\left(\frac{b^{*}}{a^{*}}\right)$$

(5) $$\mathrm{CI}=\frac{a^{*}}{b^{*}}$$

### 2.14 Microstructural properties

The microstructural properties of the samples were determined through SEM images acquired by the Cryo‐SEM technique with a Carl Zeiss 300VP unit (Carl Zeiss Microscopy LLC, White Plains, NY, USA). Prior to imaging, the pumpkin samples were freeze‐dried.

### 2.15 Instrumental textural analysis

Pumpkin desserts/jams were refrigerated at 4 ± 1°C for 24 h prior to textural analysis. The hardness values of samples were determined using a cutting apparatus (Warner‐Bratzler 60° V‐cut flat end face) attached to a texture analyzer (Shimadzu Corporation EZ‐X, Kyoto, Japan). A test speed of 5 mm/s was used, and the hardness and energy values of desserts/jams were expressed in Newton (N) and Joule (J), respectively.

### 2.16 Sensory evaluation

Sensory analyses of pumpkin dessert/jam samples were carried out according to Muñoz (1986) by 6 semi‐trained panelists between 22 and 25 years old. After refrigerating the desserts for 24 h, the samples were assigned three‐digit random codes, plated, and presented to the panelists. During training, the “round table method” was used to facilitate the interaction and calibration of panelists, whereas partitioned booths were used to avoid any interaction among panelists during actual sensory analysis. Panelists were trained by evaluating the hardness scale, which had been introduced earlier, using standard samples on a 14.5 cm scale (1: low (cream cheese), 7: medium (sausage), and 14.5: high (hard candy)). Cream cheese (Migros, MBH Food Domestic and Foreign Trade. Ltd. Company, Bursa, Turkey), sausage (Pinar, Yaşar United Marketing Distribution Tourism and Trade Inc., İzmir, Turkey), and hard candy (Olips, Kent Foodstuff Industry and Trade Inc., Company, Kocaeli, Turkey) were purchased from a local market. Training was carried out in three different sessions, each lasting 60 min on different days. Actual sensory evaluation started once the coefficient of variation of panelist scores decreased below 10%. Panelists were allowed to cleanse their palates with water (Nazli Water, Aydın, Turkey) between samples.

### 2.17 Statistical analysis

A 2×3 full‐factorial design of experiments (pre‐treatment types × pre‐treatment times) was used in this study. All experiments were conducted in triplicate. Statistical analysis of the data was performed using analysis of variance (ANOVA) with the SAS package software program (The SAS System for Windows 9.0, Chicago, USA). To identify significant differences among means, the Duncan's multiple‐range test was used at a significance level of *α* = .05. Results were expressed as the mean ± standard deviation.

## 3 RESULTS AND DISCUSSION

### 3.1 Effect of pre‐treatments on the chemical and physicochemical properties of pumpkin samples

The effect of pre‐treatment type and time on some properties of pumpkin fruit slices and desserts is presented in Table 1. ANOVA results indicated that the individual effect of pre‐treatment type on the dry matter and soluble solid contents of pumpkin fruit slices was significant (*p* < .05) but not on their ash contents (*p* > .05). When all pre‐treatment times were considered (i.e., *n* = 18), the dry matter and soluble solid contents of pumpkin fruit slices following impregnation pre‐treatments were 8.94% and 6.44%, respectively. These values increased significantly to 53.70% and 48.56% in pumpkin desserts (*p* < .05). The interaction between pre‐treatment type and time did not have a significant effect on the dry matter contents of either pumpkin fruit slices or desserts (*p* > .05). The dry matter content of untreated samples was determined to be 10.86 ± 2.14%. The insignificant effect of pre‐treatment time alone on dry matter contents could be attributed to the low calcium oxide content (0.2%–0.3%) in impregnation solution because of its low solubility in aqueous solutions. However, the incorporation of sugar led to a significant increase in the dry matter contents of pumpkin desserts in comparison to pre‐treated pumpkin slices (*p* < .05), as indicated in Table 1. The °Brix value of untreated pumpkin fruit slices was 7.67 ± 0.76%. Since the soluble solids content of impregnation solution was low, an insignificant difference was found in the soluble solids contents between the treated and untreated slices of pumpkin fruit (*p* > .05). Due to the inclusion of sucrose in pumpkin desserts, the °Brix values for dessert samples were significantly higher than those for pre‐treated slices (*p* < .05). Ash contents ranged from 0.77 ± 0.04% in untreated samples to 0.61%–0.77% in pre‐treated fruit slices on a fresh weight basis. These values were consistent with data from the USDA (2023), which reported the ash content of raw pumpkin to be approximately 0.80%. There was a statistically insignificant difference in ash contents among samples (*p* > .05). The pH value of untreated samples was 6.90 ± 2.23, and their water activity value was 0.98 ± 0.03. The effect of pre‐treatment type on the water activity of pumpkin slices was statistically insignificant (*p* > .05), but desserts (0.91 ± 0.04) had a lower water activity than pre‐treated fruit slices (0.97 ± 0.04) due to the addition of sucrose in dessert preparation (*p* < .05).

**TABLE 1:** The effect of pre‐treatment type and time on some properties of pumpkin fruit slices and desserts (mean ± standard deviation) (*n* = 3).

Sample type	Pre‐treatment type	Time (min)	Dry matter^1^ (%)	°Brix (%)	Ash^2^ (%)	pH	TPC^3^	TFC^4^	AA^5^
Pumpkin slice	Immersion under atmospheric pressure	30	9.58 ± 2.09^B^	7.50 ± 1.50^B^	0.63 ± 0.09^A^	6.97 ± 0.10^B^	22.92 ± 9.18^B^	55.79 ± 13.14^A^	17.35 ± 4.77^B^
		60	8.73 ± 1.65^B^	6.50 ± 1.80^B^	0.73 ± 0.18^A^	6.85 ± 0.16^B^	22.78 ± 9.39^B^	66.10 ± 20.17^A^	18.22 ± 5.98^B^
		90	9.26 ± 1.11^B^	6.16 ± 1.04^B^	0.64 ± 0.02^A^	6.96 ± 0.29^B^	18.83 ± 7.43^B^	88.23 ± 6.34^A^	17.36 ± 5.49^B^
	Vacuum impregnation at 100 mbar	30	9.23 ± 2.18^B^	6.17 ± 1.26^B^	0.61 ± 0.10^A^	6.86 ± 0.37^B^	21.65 ± 9.49^B^	60.33 ± 12.14^A^	18.83 ± 4.32^B^
		60	8.55 ± 1.27^B^	6.00 ± 1.00^B^	0.59 ± 0.07^A^	6.88 ± 0.40^B^	22.30 ± 9.27^B^	65.73 ± 29.32^A^	19.91 ± 5.50^B^
		90	8.29 ± 1.32^B^	6.33 ± 1.52^B^	0.63 ± 0.05^A^	6.86 ± 0.47^B^	28.03 ± 11.00^B^	69.81 ± 31.20^A^	19.87 ± 8.38^B^
Pumpkin dessert	Immersion under atmospheric pressure	30	50.07 ± 8.13^A^	48.00 ± 5.27^A^	0.71 ± 0.06^A^	5.73 ± 0.11^A^	44.70 ± 8.22^A^	22.92 ± 9.18^B^	27.33 ± 6.49^A^
		60	53.18 ± 10.55^A^	47.33 ± 7.18^A^	0.66 ± 0.08^A^	5.84 ± 0.10^A^	43.80 ± 15.34^A^	22.78 ± 9.39^B^	27.28 ± 8.90^A^
		90	47.87 ± 4.40^A^	48.33 ± 4.25^A^	0.66 ± 0.09^A^	6.05 ± 0.29^A^	49.11 ± 4.88^A^	18.83 ± 7.43^B^	34.81 ± 6.57^A^
	Vacuum impregnation at 100 mbar	30	59.85 ± 6.17^A^	51.67 ± 6.51^A^	0.66 ± 0.06^A^	5.79 ± 0.14^A^	40.62 ± 5.74^A^	21.65 ± 9.49^B^	25.58 ± 6.60^A^
		60	50.91 ± 3.41^A^	49.00 ± 3.78^A^	0.66 ± 0.16^A^	5.97 ± 0.12^A^	46.00 ± 2.73^A^	22.30 ± 9.27^B^	26.76 ± 8.07^A^
		90	54.28 ± 7.76^A^	47.00 ± 2.29^A^	0.77 ± 0.11^A^	6.46 ± 0.37^A^	38.82 ± 8.17^A^	28.03 ± 11.00^B^	26.66 ± 6.02^A^

^1^ Different superscripts within a column indicate significant differences among means (*p* < .05).

^2^ Fresh weight basis.

^3^ Total phenolic content, gallic acid equivalent per 100 g dm.

^4^ Total flavonoid content, catechin equivalent per 100 g dm.

^5^ Antioxidant activity, Trolox® equivalent per g dm.

Impregnation pre‐treatment may induce various changes in the chemical and physicochemical composition of fruits and vegetables. Jain et al. (2011) conducted an optimization study on the osmotic dehydration process for diced papaya fruits. They used solutions with different soluble solid contents (50, 60, and 70 °Brix), varied temperatures (30, 40, and 50°C), and treatment times (4.5 and 6.0 h). Their results showed that initial dry matter contents, ranging between 11.5% and 12.5%, increased to a range of 18.9% and 32.4% after osmotic dehydration. In a study by Uysal (2019), pear samples were subjected to boiling for enzyme inactivation, followed by VI under different pressures (200, 350, and 500 mbar) for various durations (15, 30, and 45 min) using impregnation solutions at 30, 40, and 50°Brix. After impregnation treatments, the highest °Brix value obtained was 18.4%, achieved with a solution at 50°Brix under 200 mbar pressure for 45 min, while the lowest (9.6%) was found for the solution at 30°Brix under 200 mbar for 15 min. Nuñez‐Mancilla et al. (2013) investigated the effect of high hydrostatic pressure (ranging from 100 to 500 MPa) applied for 10 min on strawberries during osmotic dehydration. Their results indicated that the application of high hydrostatic pressure had a significant effect on the pH values of the strawberries.

The effect of pre‐treatment type and time on the bioactive contents of pumpkin samples, such as their TPC, TFC, and AA, is also presented in Table 1. The TPC of untreated samples was determined to be 21.02 ± 8.44 mg GAE/100 g dm. When all pre‐treatment times were considered (i.e., *n* = 18), the TPC, TFC and AA values for pre‐treated pumpkin fruit slices were 22.44 ± 7.95 mg GAE/100 g dm, 67.67 ± 20.42 mg CE/100 g dm and 18.59 ± 5.06 μmol TE/g dm, respectively. Moreover, the TPC, TFC, and AA values for pumpkin desserts (*n* = 18) were 43.84 ± 8.03 mg GAE/100 g dm, 22.44 ± 8.03 mg CE/100 g dm, and 28.05 ± 6.81 μmol TE/g dm, respectively. The difference in the TPCs of samples between two pre‐treatment types was statistically insignificant (*p* > .05). Consequently, the TPCs of the untreated and pre‐treated pumpkin slices were found to be similar. In contrast, the TPCs of pumpkin desserts were found to be significantly higher in comparison to both untreated and pre‐treated slices (*p* < .05). The TFC of untreated (raw) samples was determined to be 36.77 ± 19.38 mg CE/100 g dm. The mean TFC value of all pre‐treated samples was 67.67 ± 20.42 mg CE/100 g dm. Therefore, the pre‐treatment type had a significant effect on TFC in comparison to untreated samples (*p* < .05), but its interaction with treatment time was insignificant (*p* > .05). The TFC of pumpkin desserts was lower than that of pumpkin fruit slices (*p* < .05). The interaction between pre‐treatment type and time had an insignificant effect on the total AA of pumpkin slices (*p* > .05). The AA of untreated pumpkin slice samples was 16.40 ± 4.90 μmol TE/g dm, and the mean value of all pre‐treated samples was 18.59 ± 5.06 μmol TE/g dm. Heat treatment appeared to cause an increase in the AA of slices.

The composition and structure of processed foods, along with the temperature and type of heat treatment, may contribute to an increase in the amount of phenolic compounds. In a study conducted by Blanda et al. (2008), the effect of vacuum impregnation on the properties of two different apple cultivars was investigated. The impregnation process involved using a solution containing ascorbic acid (1.00%), dextrose (37.90%), sucrose (15.20%), calcium chloride (0.25%), and sodium chloride (0.25%) under a vacuum pressure of 75 mmHg for 30 min, followed by a 5‐min restoration to atmospheric pressure. The TPCs of Stark and Granny apple cultivars (26.86% and 21.57%, respectively) were reduced by VI. In a study by Choi et al. (2006) on mushrooms, the analysis of bound and free phenolic substances, as well as AA contents, was determined with and without heat treatment. Following heat treatment at 121°C for 15 and 30 min, there was a significant increase in the amounts of both free and bound phenolics compared to untreated mushrooms. They suggested that this increase could be attributed to the release of phenolic compounds as a result of the heat treatment. In a study on the effect of the drying process on properties such as TFC, TPC, and AA values of tomato samples (Toor & Savage, 2006), there was a significant decrease in TFC, TPC, and AA values after the drying process. In a study by Türkmen et al. (2005), the effect of microwave cooking, boiling, and steaming on the AA values of pepper, broccoli, green beans, spinach, and leek samples was determined. The researchers reported that the lowest AA value (12.20%) was found in leeks while the highest increase in AA was determined in broccoli, pepper, spinach, and green beans. Gahler et al. (2003) reported that heat treatment increased the antioxidant activity of tomatoes by facilitating the release of phytochemicals such as lycopene from the food matrix. Roy et al. (2009) studied the effect of the boiling process for 5–10 min on TFC, TPC, and AA values of broccoli samples. Their findings indicated that the TFC, TPC, and AA values of boiled samples increased in comparison to fresh broccolis, suggesting a positive effect of heat treatment.

Sıçramaz and Ayar (2023) determined the influence of various pre‐processing methods on the nutritional properties of two pumpkin varieties, *Cucurbita pepo* and *C. maxima*. The TPC and AA of the freeze‐dried *C. maxima* fruits were 244.5 mg GAE and 501.2 μmol TE per 100 g dm, respectively. However, after baking, the TPC decreased to approximately 73.3 mg GAE while the AA increased to1503.2 μmol TE. In the current study, untreated fruit slices from the pumpkin variety of *C. maxima* had a TPC of about 21.02 mg GAE/100 g dm, with an AA value of about 1640 μmol TE per 100 g dm. These results may indicate that, in addition to processing methods, the pumpkin variety and its level of maturity can significantly affect the bioactive contents of the fruits.

### 3.2 Effect of pre‐treatments on the color properties of pumpkin samples

The effect of impregnation pre‐treatment type and time on the color properties of pumpkin slices and desserts, including L*, a*, b*, ∆E*, ∆C*, C*, CI and hue angle values, is given in Table 2.

**TABLE 2:** The effect of pre‐treatment type and time on the color properties of pumpkin slices and desserts (mean ± standard deviation) (*n* = 3).

Sample type	Pre‐treatment type	Time (min)	L*^1^	a*	b*	C*^2^	CI*^3^	Hue angle (°)	ΔE*^4^	ΔC*^5^
Pumpkin Slice	Immersion under atmospheric pressure	30	46.22 ± 2.26^A^	9.28 ± 3.32^A^	33.39 ± 2.61^A^	4.88 ± 2.41^A^	0.27 ± 0.08^A^	75.06 ± 4.01^A^	5.68 ± 2.89^A^	4.88 ± 2.41^A^
		60	45.81 ± 3.79^AB^	9.22 ± 3.71^A^	34.09 ± 4.29^A^	3.65 ± 3.11^A^	0.26 ± 0.08^A^	75.06 ± 4.01^A^	5.08 ± 4.50^A^	3.65 ± 3.11^A^
		90	45.59 ± 1.75^AB^	8.78 ± 4.14^A^	32.65 ± 2.49^A^	5.47 ± 3.67^A^	0.26 ± 0.11^A^	75.63 ± 5.73^A^	6.45 ± 4.14^A^	5.47 ± 3.67^A^
	Vacuum impregnation at 100 mbar	30	44.19 ± 1.70^AB^	8.31 ± 3.22^A^	32.29 ± 4.47^A^	5.97 ± 4.66^A^	0.25 ± 0.07^A^	76.20 ± 3.44^A^	7.71 ± 5.31^A^	5.97 ± 4.66^A^
		60	43.28 ± 2.29^B^	7.38 ± 4.28^A^	30.73 ± 4.85^A^	7.74 ± 5.59^A^	0.23 ± 0.10^A^	77.35 ± 5.73^A^	9.61 ± 6.37^A^	7.74 ± 5.59^A^
		90	43.97 ± 1.51^AB^	8.29 ± 3.35^A^	31.85 ± 3.22^A^	6.03 ± 3.59^A^	0.26 ± 0.08^A^	75.63 ± 4.58^A^	7.87 ± 3.86^A^	6.03 ± 3.59^A^
Pumpkin Dessert	Immersion under atmospheric pressure	30	34.39 ± 0.44^C^	5.87 ± 1.28^A^	19.18 ± 0.91^A^	20.09 ± 0.86^A^	0.31 ± 0.07^A^	72.77 ± 4.01^A^		
		60	35.90 ± 1.58^BC^	6.40 ± 1.70^A^	21.48 ± 2.60^A^	22.43 ± 2.93^A^	0.30 ± 0.05^A^	73.91 ± 2.86^A^		
		90	36.33 ± 1.07^B^	6.00 ± 1.24^A^	21.74 ± 1.06^A^	22.59 ± 0.85^A^	0.28 ± 0.07^A^	74.48 ± 3.44^A^		
	Vacuum impregnation at 100 mbar	30	37.05 ± 1.73^B^	6.53 ± 1.45^A^	20.97 ± 3.64^A^	21.98 ± 3.84^A^	0.31 ± 0.04^A^	72.77 ± 1.72^A^		
		60	36.03 ± 1.56^BC^	5.77 ± 3.23^A^	20.74 ± 2.94^A^	21.65 ± 3.58^A^	0.27 ± 0.04^A^	75.06 ± 6.88^A^		
		90	39.32 ± 1.85^A^	7.43 ± 4.74^A^	22.34 ± 5.87^A^	23.69 ± 6.98^A^	0.31 ± 0.13^A^	72.77 ± 6.88^A^		

^1^ Different superscripts within a column indicate significant differences among means (*p* < .05).

^2^ C*, chroma; CI*.

^3^ Color index.

^4^ ΔE*: Color difference.

^5^ ΔC*: Chroma difference.

The L* color value of untreated (raw) samples was determined to be 49.28 ± 2.92. The L* color values of treated pumpkin slices were lower than those of the untreated samples. There was a statistically significant difference in L* values between the two types of pre‐treatments (*p* < .05), but this difference may not be important in practical applications. There was no significant difference in L* values among the samples across different pre‐treatment times (*p* > .05). The a* and b* color values of untreated samples were 11.42 ± 2.74 and 35.61 ± 5.03, respectively. A decrease in these values was observed in pre‐treated samples, similar to their L* value. However, the a* and b* color values of the samples did not change significantly by pre‐treatment type and time (*p* > .05). The total color difference (ΔE*) of pre‐treated pumpkin slices using two pre‐treatments was found to be similar (*p* > .05). Additionally, there was no statistically significant difference between pumpkin slices pre‐treated by the two methods at different times for total chroma difference (ΔC*) values (*p* > .05). Similarly, pre‐treatment type and time did not have any statistical effect on the chroma (C*) values, color index (CI*) and hue angle values (*p* > .05). In the CIELAB color scale, a 0° hue angle value indicates a red color, and 90° indicates a yellow color. For the pumpkin samples in the present study, hue angle values ranged from 73 to 77°, indicating a slightly reddish yellow color.

Mahmud et al. (2015) conducted a study applying the immersion process to mango samples for 10 min in a solution prepared with different calcium salts (calcium chloride, calcium sulfate, and calcium ammonium nitrate). They reported that immersion did not have a significant effect on the color values of mango samples. In another study by Rodrigues et al. (2003), the effect of the osmotic dehydration process (sucrose, citric acid, or combinations of lactic acid and sodium lactate or calcium chloride) on the color values of papaya samples was investigated. The researchers reported an increase in the redness (+a*) and yellowness (+b*) values of samples, which was associated with dry matter gain. Beltekin (2019) applied several parameters, such as temperature (20–40°C), CaCl_2_ concentrations (0.50%–2.50%), and immersion time (10–60 min), to determine their effects on the sensory and physical properties of pumpkin desserts and reported an increase in the L* color values of pumpkin desserts with higher temperatures and longer immersion times. However, in the present study, the L* color values of dessert samples were lower than those of pre‐treated samples.

### 3.3 Effect of pre‐treatments on the microstructural properties of pumpkin samples

**FIGURE 2:**
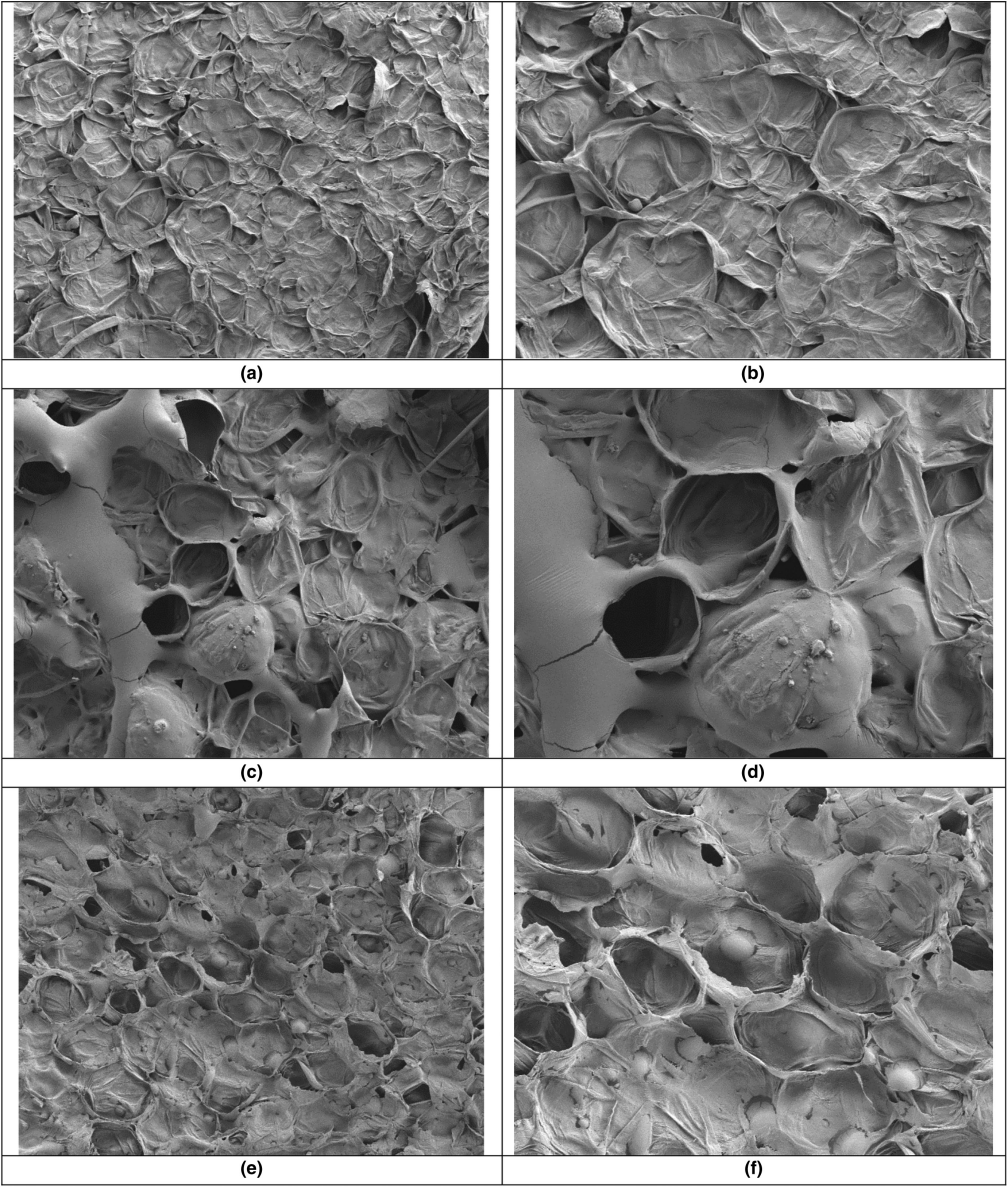
SEM images of pumpkin fruit slices that were untreated (a: 250× and b: 500×), after a 90 min‐immersion pre‐treatment (c: 250× and d: 500×) and after a 90 min‐vacuum impregnation pre‐treatment (e: 250× and f: 500× magnification).

The microstructural properties of pre‐treated pumpkin slices were determined by Cryo‐SEM analysis, and SEM images of these slices are given in Figure 2. The images of an untreated pumpkin slice are given in Figure 2a,b. The sample immersed for 90 min is presented in Figure 2c,d while Figure 2e,f indicate the images of the tissue from the sample subjected to 90 min of VI.

The SEM images of untreated samples (Figure 2a,b) indicated that pores were scarcely noticeable in plant tissues, displaying a consistent structure. In contrast, impregnation pre‐treatments lead to the formation of pores, facilitating the transition of soluble solids from the solution into tissues and the liquid transition from pumpkin slices into the solution. VI pre‐treatment was more effective than immersion, resulting in an apparent increase in calcium transfer into pumpkin slices under vacuum conditions. In SEM images of vacuum‐impregnated pumpkin slices, small particles were identified (Figure 2e,f), potentially representing calcium minerals. Unlike immersion, where pumpkin fruit slices were not subjected to vacuum, water transfer from pumpkin tissues to the solution occurred through pores in cell walls. However, the transfer of calcium from the solution into plant tissues was not as pronounced as was observed in vacuum impregnation pre‐treatment.

Safaei‐Ghomi et al. (2013) conducted a study on the structure of calcium oxide using SEM analysis, revealing that CaO was comprised of nanoparticles. In a study by Occhino et al. (2011), zucchini (*Cucurbita pepo* L.) slices were subjected to VI in a solution of CaCl_2_, using 0%–5% NaCl and 0–1 M CaCl_2_ in 10% maltodextrin solution (3:3:1, by volume). They reported an accelerated transfer of water and solutes into the samples, accompanied by the protective effect of calcium on plant tissues. In a study by Gras et al. (2003), eggplants, mushrooms, and carrots were treated by vacuum impregnation in a calcium (calcium lactate)‐enriched sucrose solution at 50 mbar for 10 min, followed by atmospheric‐pressure restoration in the solution for an additional 10 min. They reported that the calcium absorption of mushrooms and eggplants was greater compared to carrots, attributed to their porous structures. SEM analysis results showed densely packed and tightened cell tissue with impregnated calcium.

### 3.4 Effect of pre‐treatments on vitamin D3 and mineral contents of pumpkin slices

The effect of impregnation pre‐treatment type and time on the impregnated vitamin D3 contents of pumpkin fruit slices is presented in Table 3. The impregnated vitamin D3 contents were calculated by subtracting the initial vitamin D3 contents of pumpkins from the total vitamin D3 contents. The individual effect of pre‐treatment type on the impregnated vitamin D3 in pumpkin slices was found to be statistically significant (*p* < .05), and samples subjected to immersion pre‐treatment (i.e. *n* = 9) had a lower vitamin D3 content (1.95 mg/g dm) compared to those treated with VI pre‐treatment (4.09 mg/g dm) (*p* < .05). The initial vitamin D3 content in untreated pumpkin slices was determined to be 0.21 mg/g dm. A significant interaction effect between pre‐treatment type and time on the impregnated vitamin D3 contents of pumpkin fruit slices was found (*p* < .05). VI pre‐treatment was more effective than immersion in enhancing vitamin D3 impregnation in pumpkin fruit slices. When pre‐treatment type and time were considered together, impregnated vitamin D3 contents in slices increased with prolonged pre‐treatment time. The highest vitamin D3 content (6.02 mg/g dm) was found in pumpkin slices subjected to VI pre‐treatment for 90 min, although the difference from a 60‐min pre‐treatment was found to be statistically insignificant (*p* > .05). Pumpkin slices that had been pre‐treated by immersion under atmospheric pressure for 30 and 60 min had vitamin D3 contents of 1.28 and 1.85 mg/g dm, respectively (*p* > .05).

**TABLE 3:** The effect of pre‐treatment type and time on the vitamin D3 and mineral contents of pumpkin fruit slices (medium ± standard deviation) (*n* = 3).

Pre‐treatment type	Time (min)	Impregnated vitamin D3^1^ ^,^ ^2^	Impregnated calcium^3^	Potassium^3^	Magnesium^3^	Phosphorous^3^	Iron^3^
Immersion under atmospheric pressure	30	1.28 ± 0.65^C^	17.45 ± 1.78^D^	805.41 ± 48.1^A^	17.01 ± 0.65^A^	30.47 ± 9.45^A^	0.69 ± 0.49^A^
	60	1.85 ± 1.07^BC^	23.53 ± 2.61^CD^	741.22 ± 29.08^A^	21.24 ± 8.03^A^	29.33 ± 11.01^A^	2.89 ± 2.34^A^
	90	2.71 ± 0.03^BC^	30.03 ± 6.19^C^	826.73 ± 11.34^A^	18.54 ± 4.12^A^	30.03 ± 2.96^A^	2.25 ± 1.94^A^
Vacuum impregnation at 100 mbar	30	2.34 ± 0.55^BC^	43.72 ± 10.65^B^	787.94 ± 91.11^A^	25.22 ± 6.60^A^	31.16 ± 14.38^A^	0.82 ± 0.04^A^
	60	3.91 ± 1.55^BC^	64.64 ± 9.82^A^	789.33 ± 67.83^A^	18.53 ± 5.81^A^	34.27 ± 12.28^A^	1.24 ± 0.40^A^
	90	6.02 ± 1.17^A^	75.55 ± 5.24^A^	750.35 ± 49.3^A^	19.59 ± 12.10^A^	31.81 ± 8.07^A^	1.60 ± 1.14^A^

^1^ Different superscripts within a column indicate significant differences among means (*p* < .05).

^2^ Vitamin D3 contents are in mg/g dm.

^3^ Mineral contents are in mg/g ash.

The fortification of foods with vitamin D serves as a highly effective strategy to prevent vitamin D deficiency in humans. Vitamin D deficiency is prevalent because many foods may contain low levels of this vitamin. Despite being resistant to heat and cooking, both forms of vitamin D (D2 and D3) are used for fortification. Vitamin D3 form is more commonly used, and a variety of foods are fortified with this vitamin, including milk, yogurt, cheese, breakfast cereals, margarine, pastry products, and fruit juices like orange juice (Moore, 2004). The vitamin D content of foods is typically indicated in international units (IU), and 40 IU of vitamin D is equivalent to 1 mg (Nowson & Margerison, 2002). In the United States and Canada, legal maximum fortification limits are set at 90 IU for cereals and pasta, 350 IU for breakfast cereals and rice, 42 IU for milk, skimmed milk powder, and condensed milk, 100 IU for fruit juices, 331 IU for margarine, and 89 IU/100 g for yoghurts (Calvo et al., 2004). A study conducted in Australia revealed that people obtain 16% of their vitamin D intake from fortified canned fish, 10% from eggs, and 48% from margarine (Faulkner et al., 2000). These findings emphasize the significance of fortification practices in contributing to the overall vitamin D intake of the population. In a study by Park et al. (2005), apple slices of the Fuji variety were impregnated with vitamin E and minerals through the VI process at 100 mmHg pressure for 15 min, followed by exposure to atmospheric pressure for 30 min. Over a 3‐week storage period, the vitamin E content of the apple slices increased by more than 100 times, while the impregnated calcium and zinc minerals increased by about 20‐fold. The researchers reported that the inclusion of high fructose corn syrup and calcium caseinate in the impregnation solution effectively preserved the firmness of the apple slices and significantly reduced color changes after 3 weeks of storage.

According to a study by (Lacey et al., 2004), soy milk enriched with calcium, vitamin D, and chocolate flavoring offers a suitable option for individuals with lactose intolerance to meet their dietary requirements for vitamin D and calcium. The Institute of Medicine (2011) provides the recommended dietary allowance (RDA) and the tolerable upper intake level (UL) for vitamin D. The RDA values vary from 400 to 800 IU/day depending on age groups, while the UL values range from 1000 to 4000 IU/day. Commercial dietary supplements typically contain 1000–5000 IU per tablet. A portion of the pumpkin dessert produced from slices pre‐treated by the VI process (~150 g) could contribute significantly to the dietary vitamin D3 intake, falling within the range of 210–490 IU. This suggests that such a dessert/jam could be a noteworthy source of dietary vitamin D3.

The effect of pre‐treatment type and time on the mineral contents of pumpkin fruit slices is shown in Table 3. Initially, the mineral contents of untreated pumpkin fruit slices (per gram of ash) were as follows: 30.35 ± 6.43 mg Ca, 706.85 ± 32.45 mg K, 20.43 ± 9.09 mg Mg, 31.74 ± 8.25 mg P, and 1.15 ± 0.38 mg Fe, which correspond to 23.37 mg Ca, 544.27 mg K, 15.73 mg Mg, 24.44 mg P, and 0.89 mg Fe per 100 g raw pumpkin fruits. According to FoodData Central of the USDA (2023), raw pumpkins (100 g) typically contain 21.0 mg Ca, 340.0 mg K, 12.0 mg Mg, 44.0 mg P, and 0.8 mg Fe. This suggests that the mineral content of the pumpkin fruit slices was mostly consistent with the USDA data. Additionally, the figures presented in Table 3 (Impregnated Calcium) represent the calcium levels infused into fruit slices, with the initial mineral content subtracted. The individual effect of pre‐treatment type on impregnated calcium contents of pumpkin fruit slices was found to be statistically significant (*p* < .05), and samples pre‐treated with immersion (23.67 ± 6.46 mg/g ash) had a lower impregnated calcium content compared to those subjected to the VI process (58.79 ± 16.64 mg/g ash) (*n* = 9) (*p* < .05). The highest impregnated calcium content (64.64–75.55 mg/g ash) was determined in pumpkin fruit slices subjected to the VI process for 60 and 90 min, while the lowest was 17.45 ± 1.78 mg/g ash in samples pre‐treated with immersion for 30 min under atmospheric pressure. This value was found to be statistically similar to the calcium content in slices subjected to immersion pre‐treatment for 60 min (Table 3). Significant differences in impregnated calcium contents were found between pre‐treatments and among pre‐treatment times (*p* < .05). VI pre‐treatment was more effective than immersion, demonstrating an industrial potential due to reduced processing time in comparison to a traditional immersion method, which can be more time‐consuming. Moreover, it was determined that the impregnated calcium content of pumpkin fruit slices subjected to 90 minutes of the VI process was approximately 2.5 times higher than that of samples pre‐treated by immersion for the same duration. However, the effect of impregnation pre‐treatment type and time on the potassium, magnesium, phosphorus, and iron contents of pumpkin fruit slices was found to be statistically insignificant (*p* > .05). As indicated in Table 3, pre‐treated pumpkin slices had similar mineral contents with the exception of impregnated calcium.

According to the United States Department of Agriculture (USDA), 100 grams of raw pumpkin provide about 21 mg of calcium (USDA, 2023). However, this amount can vary depending on factors like the variety of pumpkin and its growing conditions. Sıçramaz and Ayar (2023) reported that freeze‐dried fruits from the *C. pepo* and *C. maxima* varieties had calcium contents of about 6 g per 100 g dm and reduced significantly for the former variety after baking. This reduction could be attributed to the release of water from plant tissues during heat treatment. In the present study, raw pumpkin fruit slices had a calcium content of about 23 mg per 100 g, while the VI pre‐treatment for 90 min increased the total calcium content of pumpkin slices to about 45 mg. These results indicate that pumpkins are not particularly high in calcium compared to some other foods, but they do contain a small amount. Indeed, VI pre‐treatment is a highly effective method for the inclusion of calcium into pumpkin fruit slices in a relatively short time.

### 3.5 Effect of pre‐treatments on the mineral contents of pumpkin desserts/jams

The effect of impregnation pre‐treatment type and time on the mineral contents of pumpkin desserts/jams is shown in Table 4. The individual effect of pre‐treatment type on the total calcium contents of pumpkin desserts/jams was found to be significant (*p* < .05), and vacuum‐impregnated samples (73.03 ± 6.26 mg/g ash) had a higher total calcium content compared to the samples subjected to immersion pre‐treatment (52.04 ± 7.14 mg/g ash) (*n* = 9) (*p* < .05). In contrast to the 3.04 mg/g ash calcium content of untreated pumpkin slices, pumpkin desserts produced from fruits subjected to VI pre‐treatments contained significantly high levels of total calcium, ranging from 67.88 to 76.13 mg/g ash.

**TABLE 4:** The effect of pre‐treatment type and time on the mineral contents and textural properties of pumpkin desserts/jams (mean ± standard deviation) (*n* = 3).

Pre‐treatment type	Time (min)	Total Calcium^1^ ^,^ ^2^	Potassium^2^	Magnesium^2^	Phosphorous^2^	Iron^2^	Sensorial hardness (cm)	Instrumental hardness (N)	Energy (J)
Immersion under atmospheric pressure	30	46.25 ± 7.27^D^	462.25 ± 21.38^A^	16.82 ± 10.39^A^	22.85 ± 4.39^A^	4.39 ± 0.23^A^	3.58 ± 1.11^C^	7.01 ± 1.25^D^	0.16 ± 0.41^D^
	60	51.41 ± 5.28^CD^	410.52 ± 51.37^A^	14.15 ± 5.55^A^	23.37 ± 5.84^A^	5.84 ± 0.34^A^	4.00 ± 1.38^C^	7.98 ± 2.19^D^	0.18 ± 0.30^D^
	90	58.45 ± 3.23^BC^	396.67 ± 45.37^A^	14.43 ± 5.70^A^	20.77 ± 1.67^A^	1.67 ± 0.16^A^	4.62 ± 1.45^C^	11.04 ± 3.26^C^	0.23 ± 0.08^C^
Vacuum impregnation at 100 mbar	30	67.88 ± 8.83^AB^	440.37 ± 42.19^A^	14.94 ± 5.73^A^	28.20 ± 7.43^A^	7.43 ± 0.34^A^	6.42 ± 0.86^B^	14.01 ± 4.37^B^	0.36 ± 0.16^B^
	60	75.07 ± 3.60^A^	425.59 ± 53.45^A^	16.46 ± 6.65^A^	26.62 ± 8.54^A^	8.53 ± 0.24^A^	7.30 ± 0.72^AB^	14.87 ± 2.62^B^	0.35 ± 0.08^B^
	90	76.13 ± 2.30^A^	412.23 ± 24.76^A^	14.48 ± 6.40^A^	23.66 ± 4.78^A^	4.78 ± 0.40^A^	8.33 ± 0.61^A^	18.92 ± 3.86^A^	0.44 ± 0.10^A^

^1^ Different superscripts within a column indicate significant differences among means (*p* < .05).

^2^ Mineral contents are in mg/g ash.

The type of pre‐treatment influenced the total calcium contents of pumpkin desserts/jams significantly (*p* < .05). In general, the total calcium content increased in both pre‐treatment types as pre‐treatment time increased, but the effect of pre‐treatment time was found to be insignificant for VI pre‐treatment (*p* > .05). Total calcium contents of desserts produced by VI pre‐treatment for 60 and 90 min were higher than those produced by immersion pre‐treatments (*p* < .05). However, differences in the potassium, magnesium, phosphorus, and iron contents of pumpkin desserts were found to be insignificant (*p* > .05). The potassium, magnesium, phosphorus, and iron contents of pre‐treated slices were lower than those of desserts, while the calcium contents of pre‐treated slices and desserts were somewhat similar when expressed on a fresh weight basis for the mineral content comparison.

Hironaka et al. (2014) conducted a study in which they impregnated 0.4 mg/g iron (ferric pyrophosphate) into potato tubers using VI under 1 kPa for 0–120 min. They reported that the iron content of potatoes subjected to VI for an hour was 6.4 times higher than that of untreated potatoes. Besides VI, high pressure can also be used for calcium infusion into foods. In a study by Gosavi et al. (2021), calcium lactate (6%) was impregnated into carrot, celery, and mango samples under high pressures ranging from 100 to 500 MPa. Under 500 MPa pressure, calcium contents were determined as 28.89 in carrots, 40.05 mg/100 g in celery, and the highest value of 111.02 mg/100 g in mango samples. Our results indicated that calcium and vitamin D3 can be successfully infused into pumpkin fruit slices by the VI process under 100 mbar. This suggests the potential for the effective impregnation of beneficial nutrients into food products using this process.

### 3.6 Effect of pre‐treatments on the textural properties of pumpkin desserts

Fortifying pumpkin fruit slices with calcium and vitamin D3 through an impregnation pre‐treatment did not have an adverse effect on the taste of the dessert samples. In our preliminary experiments, the use of CaCl_2_ solution resulted in a bitter taste in pumpkin desserts. Therefore, CaO was more suitable for calcium fortification in pumpkin fruit slices instead of CaCl_2,_ as it did not influence the taste of pumpkin desserts.

Following impregnation pre‐treatments, the pumpkin slices were cooked using a mixture of sugar and water to produce desserts. The sensory hardness (cm), textural hardness (N), and energy values (J) of the desserts were then determined, and the effect of pre‐treatment type and time on the sensorial and instrumental textural properties of pumpkin desserts is presented in Table 4.

The difference in the sensory hardness values of pumpkin desserts between pre‐treatment types was found to be statistically significant (*p* < .05). The sensory hardness of desserts produced with vacuum‐impregnated slices for 90 min was determined to be 8.33 ± 0.61 cm, and it was similar to the hardness of a sample pre‐treated by VI for 60 min (*p* > .05). Lower hardness values were determined in samples pre‐treated by immersion, ranging between 3.58 and 4.62 cm. In the case of samples pre‐treated with VI, a statistically significant difference was found between samples pre‐treated for 30 and 90 min (*p* < .05). Among the pre‐treatments, VI for 90 min yielded the most effective result in terms of sensory hardness. It was observed that both the pre‐treatment type and time played crucial roles in determining the sensory hardness of the desserts.

Statistically significant differences in instrumental hardness and energy values were also found among pumpkin desserts (*p* < .05). VI pre‐treatment again proved more effective in terms of instrumental hardness than immersion under atmospheric pressure. The hardest sample (18.92 ± 3.86 N) was the one produced with slices pre‐treated under 100 mbar pressure for 90 min. Samples pre‐treated with VI for 30 and 60 min had statistically similar instrumental hardness values (*p* > .05). The softest samples were those immersed under atmospheric pressure for 30 and 60 min. Both hardness values and energy applied to samples followed a similar pattern.

In a study by Beltekin (2019), immersion pre‐treatment in a calcium chloride solution (0.50%–2.50%) was applied to pumpkins at temperatures ranging from 20 to 40°C for holding times between 10 and 60 min. The hardness values of pumpkin desserts varied from 15.00 to 29.67 N, while the hardness value of an untreated sample (control) was 12.00 N. This study reported that an increase in temperature and a shorter holding time led to a decrease in the hardness value of the desserts. Furthermore, holding time was found to be more influential than temperature in affecting hardness. Additionally, the hardness of the desserts increased with an increase in calcium chloride concentration. Beltekin (2019) also reported the sensory characteristics of pumpkin desserts, where the control samples had a sensory hardness value of 1.29 ± 0.49, a color rating of 2.86 ± 0.38, a flavor rating of 1.86 ± 0.69, and an overall acceptability score of 2.57 ± 0.53. In contrast, the pre‐treated pumpkin desserts had the highest sensory hardness value (4.00 ± 0.58). Moreover, among the pumpkin desserts, the sample pre‐treated by immersion with the lowest CaCl_2_ concentration (0.50%) was perceived as the most delicious, while the one pre‐treated with the highest CaCl_2_ concentration (2.50%) was evaluated as the least tasty. These findings highlight the impact of calcium chloride pre‐treatment on both the physical and sensory attributes of pumpkin desserts. In our study, the sensory hardness value of samples pre‐treated by immersion was between 3.58 and 4.62 cm (sausage: 7 cm). Our preliminary studies involving impregnation pre‐treatments with calcium chloride revealed a noticeable bitter taste in pumpkin desserts. This aligns with the findings reported by Beltekin (2019), where an increase in CaCl2 concentration in impregnation solutions led to an undesirable taste in the desserts. The bitter taste observed in pumpkin desserts with higher CaCl_2_ concentrations in impregnation solutions could be a contributing factor to the perceived undesired taste. Jan et al. (2016) investigated the effect of calcium salts on the softness and physicochemical properties of apples during storage, and reported that the hardness values of apples were significantly influenced by the immersion process in a calcium chloride solution (9%) for 12 min. Moreover, tissue hardening in apples was reported as a result of calcium impregnation. In a study by Anjum and Ali (2004), mango samples were immersed in a solution of calcium chloride (2.5%), calcium sulfate (5.0%), or calcium ammonium nitrate (7.5%). They reported that fruit ripening was delayed due to the increased concentration of calcium salts after processing, but there was shrinkage on the surface of mango fruits. From a sensory standpoint, as the concentration of calcium salts increased, the flavor of mango decreased, and bitterness was noted. These findings highlight the influence of calcium salts on the texture, ripening, and sensory attributes of fruits. In a study conducted by Degraeve et al. (2003), calcium chloride (CaCl_2_) and pectin methyl esterase (PME) were impregnated into apple, strawberry, and raspberry fruits. The impregnation was achieved using CaCl_2_.2H_2_O (1%) and PME (0.6%) (1:1, v/v) in a sucrose solution (35%) under 50 mmHg pressure for 2 min. They found that the tissues of fruits exposed to PME and CaCl_2_ solutions were successfully hardened as a result of the impregnation process. In the present study, the VI with CaO solution at 100 mbar was found to increase both sensory and textural hardness values of pumpkin desserts with an increase in pre‐treatment time, as indicated in Table 4. This suggests that the VI process had a positive effect on the hardness characteristics of the pumpkin desserts, aligning with the observed trends in sensory and textural properties.

## 4 CONCLUSIONS

Traditional production of pumpkin desserts/jams with hardened outer layers typically takes a very long time in small‐ and medium‐sized enterprises in the food industry. The goal of this study was to reduce this time with the use of VI pre‐treatment, a method that may be more suitable for industrial applications. The CaO in the impregnation solution was used to harden pumpkin fruit slices in terms of their texture, similar to the traditional method. The impregnation pre‐treatment type and time changed the dry matter and ash contents, °Brix values, the total phenolic content, TFC, and antioxidant activity of pumpkin slices significantly. However, both the instrumental and sensorial hardness of pumpkin desserts increased with an increase in VI pre‐treatment time. This suggests that while the VI process did not have a notable impact on certain chemical properties of the pumpkin fruit slices, it did influence the texture of the resulting desserts/jams, making them harder as the pre‐treatment time with VI increased. The pre‐treatment of VI was approximately 2 times more effective than immersion in terms of vitamin D3 infusion into pumpkin fruit slices. On average, the former transferred approximately 2.5 times higher calcium than the latter pre‐treatment. Results showed that the mass transfer of calcium and vitamin D3 into pumpkin fruit slices was more efficient and faster with the use of VI pre‐treatment compared to immersion. Calcium in the impregnation solution played a crucial role in the formation of hardened tissues around pumpkin desserts. Furthermore, the impregnated vitamin D3 reached a level that could make a significant contribution to the daily dietary requirement of this vitamin for an adult. Overall, VI proved to be a more effective method for the infusion of both calcium and vitamin D3 into pumpkin fruit slices in comparison to immersion.

The results of this study suggest that the time required for the traditional production of pumpkin desserts/jams could be significantly reduced from 48 h to about 3 h, achieved through 90 min of VI followed by 85 min of cooking. This demonstrates the potential of VI pre‐treatment for the industrial production of pumpkin desserts/jams. While the contribution of the calcium impregnated into pumpkin desserts/jams may be limited for dietary calcium intake due to the low solubility of calcium oxide in water, the impregnated calcium may play a crucial role in creating desirable textural characteristics in pumpkin desserts/jams without any adverse effects on their sensory properties. This highlights the efficiency and effectiveness of VI as a method to streamline the production process of pumpkin desserts/jams in an industrial setting.

## AUTHOR CONTRIBUTIONS

**Elif Buse Taş:** Conceptualization (equal); data curation (equal); formal analysis (equal); writing – original draft (equal). **Oguz Gursoy:** Conceptualization (equal); data curation (equal); methodology (equal); writing – review and editing (equal). **Yusuf Yilmaz:** Conceptualization (equal); data curation (equal); methodology (equal); supervision (equal); writing – original draft (equal); writing – review and editing (equal).

## CONFLICT OF INTEREST STATEMENT

The authors state no conflict of interest.

## ETHICS STATEMENT

The research conducted is not related to either human or animal use.

## Data Availability

The datasets generated during and/or analyzed during the current study are available from the corresponding author on reasonable request.
